# Impact of surgery on quality of life of Ugandan women with symptomatic pelvic organ prolapse: a prospective cohort study

**DOI:** 10.1186/s12905-021-01397-z

**Published:** 2021-06-25

**Authors:** Musa Kayondo, Dan Kabonge Kaye, Richard Migisha, Rodgers Tugume, Paul Kalyebara Kato, Henry Mark Lugobe, Verena Geissbüehler

**Affiliations:** 1 Faculty of Medicine, Mbarara University of Science and Technology, P.O.BOX 1410, Mbarara, Uganda; 2 Department of Obstetrics and Gynecology, Mbarara Regional Referral Hospital, P.O.BOX 40, Mbarara, Uganda; 3 Department of Obstetrics and Gynecology, Makerere University College of Health Sciences, Kampala, Uganda; 4 Department of Physiology, Mbarara University of Science and Technology, P.O.BOX 1410, Mbarara, Uganda; 5 Department of Gynecology, St. Claraspital, Basel, Switzerland

**Keywords:** Mbarara, Impact, Pelvic organ prolapse, Quality of life, Surgery

## Abstract

**Background:**

Pelvic organ prolapse (POP) is a significant public health issue that negatively affects the Quality of Life (QOL) of women in both low and high-income countries. About 20% of women will undergo surgery for POP over their lifetime. However, there is a paucity of information on the effect of surgery on QOL especially in resource-limited settings. We therefore assessed the QOL among women with symptomatic POP living in rural southwestern Uganda and the impact of surgery on their quality of life.

**Methods:**

We conducted a prospective cohort study among 120 women with symptomatic POP scheduled for surgery at the urogynecology unit of Mbarara Regional Referral Hospital. The QOL at baseline and at 1 year after surgery in the domains of physical performance, social interaction, emotional state, sexual life, sleep quality, personal hygiene and urinary bladder function was determined using a King’s Quality of Life questionnaire. A paired t-test was used to compare the difference in mean scores at baseline and at 1-year post-surgery.

**Results:**

Of the 120 participants that were enrolled at baseline, 117(98%) completed the follow-up period of 1 year. The baseline QOL was poor. The domains with the poorest QOL were physical, social, sexual, emotional and sleep quality. The mean QOL scores in all the domains and the overall QOL significantly improved 1 year after surgery (p < 0.001). The overall QOL improved by 38.9% after surgery (p < 0.001).

**Conclusions:**

The QOL was poor among women with symptomatic POP and surgery improved the QOL in all the domains of life. We recommend that surgery as an option for treatment of symptomatic POP should be scaled up to improve on the QOL of these women.

## Background

Pelvic Organ Prolapse (POP) is defined as an anatomic support defect of the pelvic viscera resulting from the long-term failure of their supporting and suspension mechanisms [1]. This weakness in the supporting mechanisms leads to descent of the pelvic organs, including the bladder, uterus, rectum and or small intestines, into or outside of the vagina [2]. POP affects more than half of parous women over the age of 50 years worldwide [3]. Various risk factors for POP have been reported including childbirth, ageing, heavy manual labour and menopause [4].

POP affects many aspects of women’s life including physical, psychological, social interaction, sexual function and hygiene [5]. Women with POP present with a variety of symptoms including urinary, bowel, sexual, and psychological which greatly compromise their quality of life (QOL) [6–9]. In the advanced stages, POP presents with debilitating physical symptoms like difficulty in walking, sitting and squatting, which negatively impacts the daily economic activities of these women like farming eventually resulting in poverty [10, 11]. In addition, women with POP frequently report limitations to their sexual life such as: lack of sexual desire, arousal, orgasm, and pain during intercourse which ultimately leads to loss of sexual interest with some being abandoned by their husbands [5, 7, 12–14]. A number of psychological and mental health problems have been reported among women with POP including emotional disturbances, depression, loss of self-esteem, lack of sleep, rejection and isolation [15–18].

Therefore, symptomatic POP requires management which may be surgical or nonsurgical (conservative) to improve QOL. Conservative treatment options like pessaries, lifestyle change, and pelvic floor exercises have been shown to be effective as an alternative to surgery [19–21]. However, pessaries are not readily available and hence their use by clinicians in treatment of symptomatic POP in this setting is low [22], leaving surgery as the main mode of management of symptomatic POP.

About 20% of women will undergo surgery for POP over their lifetime [23] with close to 200,000 POP surgeries being performed in the United States of America (USA) annually [24]. However, access to POP surgery in low income countries is very limited because it is expensive and the skilled personnel to perform the surgery are few [25]. Surgery for symptomatic POP has been shown to improve the QOL in high income countries [5, 13, 26–28]. However, in low income countries including Uganda, the impact of surgery on QOL is rarely reported yet this evidence is important for advocacy in scaling up treatment for POP in these resource limited settings where women with POP face various challenges in accessing care [12].

Therefore, in this study we aimed to assess the quality of life among women with symptomatic POP in rural Southwestern Uganda before and after surgery in the seven life domains; physical, social interaction, psychological, sexual activity, personal hygiene, sleep and bladder function.

## Methods

### Study setting, design and study population

We conducted a prospective cohort study at the Urogynecology unit of Mbarara Regional Referral Hospital (MRRH) from December 1, 2018 to December 31, 2020, among women diagnosed with symptomatic pelvic organ prolapse (POP) scheduled for surgery. MRRH is a tertiary Hospital located in Mbarara district in Southwestern Uganda about 250 km from the capital city of Kampala. MRRH is the main referral hospital of the entire southwestern Uganda serving over 10 districts and also gets patients from the neighboring countries of Tanzania, Rwanda, Burundi and the Eastern Democratic Republic of Congo (DRC).

### Data collection

#### Preoperative workup

The diagnosis of POP, clinical exam, staging of POP, and decision to do surgery was made by the urogynecology surgical team. Participants were diagnosed to have POP if they had any one of the following clinical findings: cystocele, urethrocele, cystourethrocele, uterine prolapse, vault prolapse, enterocele, or rectocele. Staging of POP was done using the Pelvic Organ Prolapse Quantification (POP-Q) system validated by the International Continence Society (ICS) into stages I, II, III and IV [29, 30].

An interviewer guided data capture tool was administered to collect information on the baseline characteristics of the study participants. These included: age, parity, education level, marital status, occupation, smoking, alcohol use, type and severity of the prolapse. Age in years was categorized according to reproductive age groups: 18–34 (early reproductive age group), 35–49 (late reproductive), 50–59 (peri-menopausal) and ≥ 60 (post-menopausal).

The participants had their quality of life (QOL) determined at enrolment prior to surgery. The QOL was determined using the King’s Health Questionnaire [31]. This questionnaire was validated to assess the QOL among women with urinary incontinence but we used it to assess QOL among women with POP in our study as a number of women with POP have been shown to have urinary incontinence [32–34]. This interviewer-based questionnaire assesses seven QOL domains that include physical/daily roles performance, social interraction, sexual function, emotional/psychological state, personal hygiene, sleep quality and bladder function. A score (%) for each of the domains was calculated. Each life domain had a score ranging from 0 to 100%. The overall QOL for each participant was obtained as an average of the total scores in the seven domains. The higher the scores the poorer the QOL. Trained research assistants who included counsellors and nurses who were not part of the surgical team conducted the interviews and completed the King’s Heath Questionnaire.

#### Surgery for pelvic organ prolapse

The participants underwent surgery for the management of POP. Surgery was performed in those who had symptomatic POP stage II, III and IV and was dependent on the type of prolapse. The different surgical procedures that were performed include anterior colporrhapy for cystocele, posterior colporrhapy for rectocele, vaginal hysterectomy with vaginal vault suspension (sacrospinous ligament or uterosacral vault suspension) for uterine prolapse in those who had completed child bearing and did not want uterine sparing surgery, and bilateral sacrospinous vault suspension in those with recurrent vaginal vault prolapse. Cervicopexy was done for those with uterine prolapse that hadn’t completed child bearing or wanted uterine sparing surgery.

#### Postoperative follow up

After discharge, the participants were invited back to the hospital one year after surgery for a follow-up visit. Participants were contacted through a phone call one week prior to their scheduled postoperative visit. The purpose of the call was to remind the participants of their scheduled follow-up visit. Participants who could not be reached on phone were traced using the contacts of their next of kin. This was to minimize loss to follow-up. At this follow-up visit, the QOL one year after surgery was again assessed by the trained research assistants using the King’s Health Questionnaire.

### Power calculation

A post-hoc power calculation was done where we assumed a sample size of 120, mean QOL score before surgery of 44.5 with a standard deviation of 20.9 and a mean QOL score after surgery of 8.0 with a standard deviation of 11.6. We therefore obtained a power of 100% to detect a difference in means. This sub study was part of a larger study to determine the recurrence rate of POP following surgery.

### Statistical analysis

Data were entered into Redcap and exported to Stata 13 (StataCorp, LLP, College Station, TX, USA) for analysis. Categorical data were presented as frequencies (%). The mean QOL score and the 95% confidence intervals for each of the seven domains and the overall mean QOL at baseline and at 1 year after surgery were calculated. The overall mean QOL score before and at 1 year after surgery was compared as well as the mean scores in each of the domains. A paired t-test was used to determine if there was a significant difference in the means. A p value of < 0.05 was considered significant.

### Ethical considerations

Ethical approvals were obtained from the Mbarara University of Science and Technology (MUST) Research Ethics Committee and the Uganda National Council for Science and Technology (UNCST) number HS368ES. Written informed consent was obtained from all the study participants or from their legally authorized representatives for those who couldn’t read or write. Confidentiality was observed during all the interviews. Personal identifiers such as name and in-patient number were not collected. The study participants were assigned study ID numbers.

## Results

A total of 130 women with symptomatic POP scheduled for surgery were seen during the study period. Of these, 10 declined to consent and were not included in the study. We therefore enrolled a total of 120 participants into the study. The participants were followed up for 12 months post-surgery, with 117 completing the follow-up period giving a completion rate of 97.5%. The mean age of the study participants was 55 years (SD ± 15) with the majority (n = 46, 38.3%) aged ≥ 60 years, peasant farmers (n = 106, 88.3%) and had had five or more deliveries (n = 94, 78.3%) as shown in Table 1.

**Table 1:** Baseline participants’ characteristics

Characteristic	Description	Total (N = 120)	
		Frequency	Percentage
Age in years	18–34	7	5.8
	35–49	35	29.2
	50–59	32	26.7
	≥ 60	46	38.3
Residence	Rural	115	95.8
	Urban	5	4.2
Level of education	None	65	54.2
	Primary	49	40.8
	Secondary	4	3.3
	Tertiary	2	1.7
Marital status	Married	59	49.2
	Separated	12	10.0
	Single	32	26.7
	Widowed	17	14.2
Occupation	Peasant	106	88.3
	Business	9	6.9
	Others	8	6.7
Monthly income^φ^	< 50 k	84	70.0
	50–100 k	25	20.8
	> 100 k	11	9.2
Parity	0–4	26	21.7
	≥ 5	94	78.3
History of smoking	No	82	68.3
	Yes	38	31.7
Alcohol consumption	No	67	55.8
	Yes	53	44.2

^φ^Income in Ugandan shillings; 1 k = 1000 shillings

The majority of the participants had uterine prolapse (n = 85, 70.8%) and the commonest stage of POP was stage III (n = 56, 46.7%) as shown in Table 2.

**Table 2:** Types and stages of POP among the study participants

Characteristic	Description	Total (N = 120)	
		Frequency	Percentage
Anterior vaginal wall prolapse	No	33	27.5
	Yes	87	72.5
Posterior vaginal wall prolapse	No	88	73.3
	Yes	32	26.7
Uterine prolapse	No	35	29.2
	Yes	85	70.8
Post hysterectomy Vault prolapse	No	117	97.5
	Yes	3	2.5
Enterocele	No	113	94.2
	Yes	7	5.8
Pre-surgery POPQ stage	I	0	0.0
	II	28	23.3
	III	56	46.7
	IV	36	30.0

The surgical procedures that were performed and the related complications are shown in Table 3. The most common procedure performed among the study participants was Vaginal hysterectomy with sacrospinous ligament vault fixation in 66 (55%) of the participants. Intraoperative complications were encountered in four patients and these included: rectal injuries (n = 2), hemorrhage that required blood transfusion (n = 1), and urinary bladder injury (n = 1). The main postoperative complication was vaginal cuff infection which occurred in 10 (8.3%) of the participants.

**Table 3:** Surgical procedures performed among the study participants and complications

Surgical procedures n = 120	n (%)
Vaginal hysterectomy with sacrospinous ligament vault fixation	66 (55)
Vaginal hysterectomy plus anterior and posterior repair with uterosacral ligament vault fixation	17 (14.2)
Posterior colporrhaphy only	15 (12.5)
Anterior colporrhaphy only	13 (10.8)
Bilateral sacrospinous vault fixation for vaginal vault prolapse	3 (2.5)
Anterior and Posterior Colporrhaphy combined	3 (2.5)
Sacrospinous Cervicopexy	3 (2.5)
Intraoperative complications	4 (3.3)
Postoperative complications	10 (8.3)

The baseline mean QOL overall and in the specific life domains is shown in Table 4. The participants presenting with POP had a poor QOL with a mean score at baseline of 45.5% (95% CI; 41.7–49.3%). High mean QOL scores were found in the physical, social, emotional, sexual and sleep domains ranging from 40.4 to 61.9%. Personal hygiene and urinary bladder function had the lowest mean QOL scores of 19.9% (95% CI; 15.1–24.8%) and 13.6% (95% CI; 10.1–17.2%) respectively.

**Table 4:** Baseline mean QOL scores compared to mean QOL scores at 1 year after surgery for POP

Quality of life domains	Baseline	One-year post-surgery	
	Mean %(95%CI)	Mean% (95%CI)	*P* value
Overall	45.5 (41.7–49.3)	6.6 (4.5–8.8)	< 0.001
Physical	66.7 (60.0–73.3)	11.6 (8.1–15.2)	< 0.001
Social	61.9 (55.6–68.1)	9.0 (5.9–12.1)	< 0.001
Emotional	58.4 (52.3–64.6)	5.6 (2.6–8.5)	< 0.001
Sexual	69.1 (61.0–77.3)	13.5 (8.2–18.9)	< 0.001
Sleep	40.4 (34.6–46.2)	4.9 (1.7–8.1)	< 0.001
Hygiene	19.9 (15.1–24.8)	0.5 (0.3–1.7)	< 0.001
Bladder function	13.6 (10.1–17.2)	4.6 (2.7–6.5)	< 0.001

There was a significant improvement in the overall quality of life as well as in the different domains 1 year after surgery for symptomatic POP as shown in Table 4. The overall mean QOL score at baseline of 45.5% decreased by 38.9–6.6% at 1 year after surgery and this change was statistically significant (p < 0.001). There was also a decrease in all the specific QOL domains at 1 year after surgery for POP. Physical activity domain decreased by 55.1% from 66.7 to 6.6%, social interaction decreased by 52.9% from 61.9 to 9%, emotional status reduced by 52.8% from 58.4 to 5.6%, sexual performance by 55.6% from 69.1 to 13.5% and sleep quality also decreased by 35.5% from 40.4 to 4.9%. Though low at baseline, the mean scores for personal hygiene and urinary bladder function also decreased at 1 year post-surgery by 19.4 and 9% respectively. All these changes were statistically significant (p < 0.001).

## Discussion

This study showed that women presenting with symptomatic POP had a poor quality of life before surgery with an overall mean QOL score of 45.5%; surgical management for the symptomatic POP significantly improved the QOL by 38.9%.

The poor QOL in this study is similar to findings from studies done in China and rural Pakistan [35, 36]. This is probably because POP is a complex condition which affects both physical and functional aspects of a woman including physical, social, emotional, sleep, and sexual performance ultimately leading to poor QOL [5].

The poor physical performance observed in this study was also reported in studies done in other low income countries [10, 11, 37]. A study done in South Africa by Brandt et al. 2019 suggested that the poor physical performance in women with POP could be due to chronic pelvic pain caused by stretching and weakening of the pelvic ligaments which makes it difficult for these women to walk, bend and work. Women with POP in our study also had a poor social life score. This is similar to what was observed in studies done in Nepal and Ethiopia [12, 37, 38]. This could be due to the fact that advanced stages of POP (III and IV) are associated with foul smelling vaginal discharge and urinary incontinence which limits the interaction with the community for fear of being discriminated [39]. The poor QOL score among women with POP in the emotional domain was similar to what was found in a study done in USA [16]. This low emotional state in women with POP could be due to a combination of perceived decreased body image, shame, and non-disclosure for fear of discrimination or even divorce in these women [12, 40]. Among the women who were still sexually active, the score in their sexual life was poor and this is similar to what was reported in other studies [5, 7, 12–14]. Women with advanced POP are likely to experience dyspareunia, lack of libido and arousal ultimately leading to poor sexual performance [7, 12]. The participants also had poor QOL score in the sleep domain as was found elsewhere [16]. The poor sleep quality may be attributed to the frequent nocturia that is common in women with symptomatic POP [41]. Additionally, the loss of sleep could be as a result of depression in these women [16].

One year following surgery, the overall quality of life including physical, social, emotional, sleep and sexual performance improved. This is similar to what was observed in studies done in the high and low income countries where surgery significantly improved all aspects of women’s life [5, 7, 13, 14, 37, 38, 41]. Bilateral sacrospinous ligament vault suspension which was performed for participants with recurrent vaginal vault prolapse in this study has been shown to improve QOL [42, 43]. Surgery may have improved the overall QOL of life because it relieves patients’ symptoms, restores normal anatomy and function of the pelvic structures [44].

The improvement in physical performance after surgery is similar to that observed in a study from Ethiopia [38]. This improvement could be due to the correction of the anatomical defect leading to a reduction in the chronic pain and discomfort which enables the women to walk and even work comfortably [37]. The social life of the women similarly improved probably because POP surgery has been found to correct associated urinary incontinence [45] which ultimately takes away the bad urine smell resulting in improvement in the social domain [37, 46]. The QOL in the emotional domain improved after surgery similar to that reported in a previous study among American women [41]. The improvement may be because the women did not have any more worries about their body image perception and fear about discrimination from society after surgery [41, 47]. The sleep domain improved after surgery as in a study by Ghetti et al. [41]. This improvement is probably because of the improvement in bladder symptoms such as nocturia as well as improvement in depressive symptoms which occurs after surgery [41, 45, 48, 49]. As was reported in other studies from the developed world [5, 7, 50], sexual function improved after surgery. This is probably because the restoration of the normal vaginal length after surgery leads to a reduction in the dyspareunia that used to be experienced by these women [12, 13, 47]. Additionally, this improvement could be due to a related improvement in the emotional status [16]. Therefore, in addition to surgery, women with symptomatic POP would benefit from a multidisciplinary approach for accurate management [9, 51]. These women should be offered psychosocial support and counselling because they have emotional, social and sexual problems.

Our study has some limitations. This study was conducted at a single regional referral hospital in southwestern Uganda and the findings may not be generalizable to all other women in Uganda. The King’s Health Questionnaire was validated to assess the QOL among women with urinary incontinence but we used it to assess QOL among women with POP in our study since a significant number of women with POP also have urinary incontinence.

## Conclusions

Quality of life among women with POP was poor overall and surgery greatly improved women’s quality of life. Surgery, as an option for the management of POP, should be scaled up to improve the QOL among women with symptomatic POP. In addition, women with symptomatic POP should be offered psychosocial support and counselling because they have emotional, social and sexual problems.

## Data Availability

The datasets used and/or analyzed during the current study are available from the corresponding author on reasonable request.

## Abbreviations

POP: Pelvic organ prolapse
PFDs: Pelvic floor disorders
QOL: Quality of Life
POP-Q: Pelvic organ prolapse quantification
ICS: International Continence Society
USA: United States of America
MRRH: Mbarara Regional Referral Hospital
MUST: Mbarara University of Science and Technology
UNCST: Uganda National Council of Science and Technology
